# Preparing Uniform-Thickness Corneal Endothelial Grafts from Donor Tissues Using a Non-Amplified Femtosecond Laser

**DOI:** 10.1371/journal.pone.0083185

**Published:** 2013-12-05

**Authors:** Kanwarpal Singh, Nour Haydari, Isabelle Brunette, Santiago Costantino

**Affiliations:** 1 Centre de Recherche de l'Hôpital Maisonneuve-Rosemont, Montréal, Québec, Canada; 2 Department of Ophthalmology, Faculty of Medicine, Université de Montréal, Montréal, Québec, Canada; 3 Biomedical Engineering Institute, Université de Montréal, Montréal, Québec, Canada; University of Missouri-Columbia, United States of America

## Abstract

Corneal grafts for Descemet’s Stripping Automated Endothelial Keratoplasty are commonly prepared using mechanical microkeratomes. However, the cuts produced in such way render corneal lenticules that are thinner centrally than peripherally, thus inducing a hyperopic shift. Here we describe a novel device for preparing donor corneal grafts, in which a single low-energy femtosecond laser system is used as both a light source for optical coherence tomography and for cutting the graft illuminating from the endothelial side. The same laser is first utilized to obtain three-dimensional optical coherence tomography images of the donor tissue for guiding the dissection and obtaining grafts of uniform thickness with no applanation or contact. This device allows an optimal procedure for preparing consistently thin posterior grafts for transplantation.

## Introduction

The newer techniques that have been developed for corneal transplantation are limited to grafting only the diseased layers, while leaving the host’s healthy corneal layers untouched. Descemet’s Stripping Automated Endothelial Keratoplasty (DSAEK) [1,2] recently became the gold standard technique for endothelial keratoplasty. The host cornea is prepared using a standardized microkeratome to cut the anterior cornea and retain the posterior stroma with Descemet’s Membrane and the Endothelium.

The functional outcome of DSAEK is partly limited by sub-optimal optical results, which are directly related to the shape of the graft. Grafts routinely prepared with mechanical microkeratomes are typically thinner centrally than peripherally, yielding a hyperopic shift (negative lens effect) that necessitates additional optical correction in the spectacles of the operated eye. The mean postoperative hyperoptic shift is +1.25D, with inter-subject variability in refraction in the order of 4.0 to 5.0 D [3-12]. In order to obtain more uniform and ultra-thin DSAEK lenticules, double-pass microkeratome cuts have been proposed, where the second cut starts from the end of the first cut [13]. This technique, however, is still overshadowed by a non-negligible rate of microkeratome-related complications (7.2 %) occurring during donor tissue preparation [14].

In an attempt to further improve the optical quality of the grafted eye, Descemet’s membrane endothelial keratoplasty (DMEK)[15] was proposed, a surgical variant where the corneal endothelium along with its Descemet’s membrane are manually detached from the donor cornea and transplanted. DMEK provides better visual outcome than DSAEK, but it is technically more challenging, even in the hands of the most experienced surgeons [16]. There is no clear consensus on the optimal thickness of the endothelial graft since there is a tenuous relationship between the graft thickness and visual acuity [17-19]. Femtosecond lasers have been used to prepare corneal grafts by making planar cuts in the cornea [20], which leads to graft of uneven thickness, i.e. thicker at the edge and thinner in the center. A technique for preparing uniform thickness graft using a femtosecond laser (FSL) has also been described [21] in which the cornea is applanated from its endothelial side.

The ideal endothelial graft must have a uniform profile to avoid aberrations and should be thick enough to ease manipulation [22]. We propose here a new instrument for preparing such donor corneal grafts, in which a low-energy FSL system is used as both a light source for optical coherence tomography (OCT) [23-25] and for cutting the graft illuminating from the endothelial side. In our technique, the same laser is utilized to obtain three-dimensional (3D) OCT images of the donor tissue for guiding the dissection and obtaining grafts of uniform thickness with no applanation or contact. We believe this device allows an optimal procedure for preparing consistently thin posterior grafts for transplantation. 

## Materials and Methods

**Figure 1:**
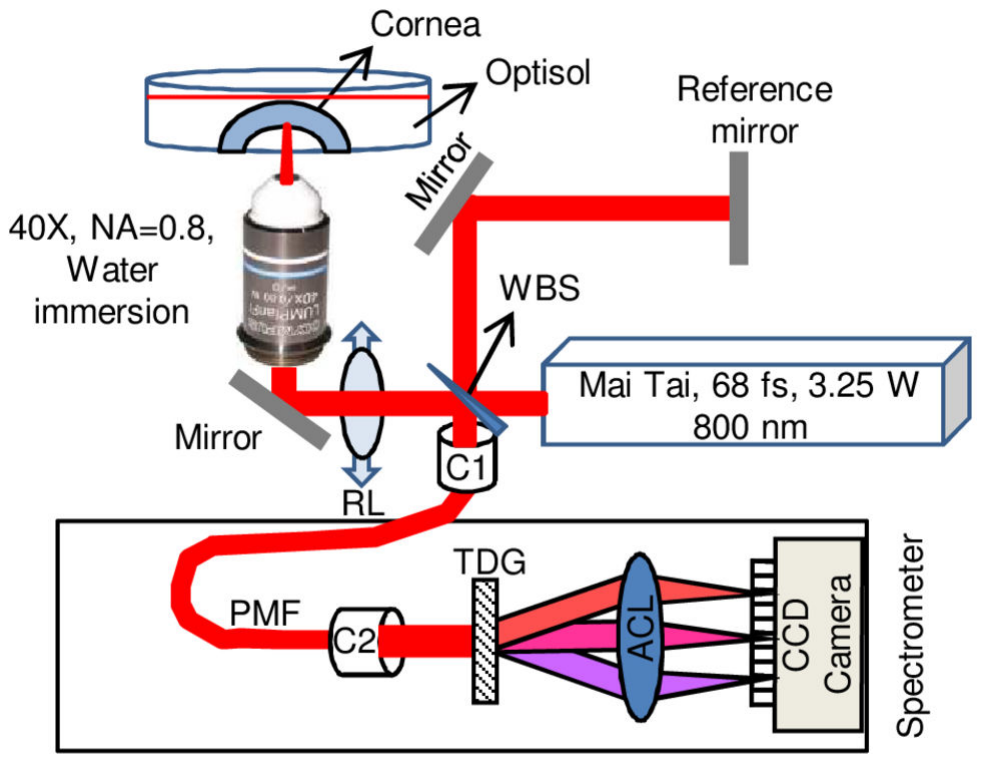
Schematic of the optical system used for corneal dissection and imaging. The abbreviated components are: movable lens (ML), polarization maintaining fiber (PMF), collimator (C1, C2), transmission diffraction grating (TDG), achromatic lens (ACL), wedge beam splitter and charged coupled device camera (CCD camera) .

All experiments were conducted in accordance with the Declaration of Helsinki. The schematic of the experimental system used in this work is shown in Figure 1. The beam of a non-amplified femtosecond laser (Mai Tai, Newport) operated at 800 nm wavelength was divided using a wedge beam splitter. The reflected and transmitted beams were directed towards a reference mirror and the cornea, respectively. The same laser system was operated in two modes: OCT *imaging mode* [25-27] and corneal *photo-disruption mode*, by only varying power. 

In *imaging mode*, the laser beam was focused at the cornea placed in a glass-bottom petri dish filled with Optisol-GS (Bausch and Lomb, Rochester, NY), through the combination of a removable lens (RL, f=100 mm) and a high numerical aperture (NA=0.8, working distance = 3.3 mm) water immersion objective (Olympus, Tokyo, Japan). The removable lens was sometimes used to increase the overall depth of focus at the cornea (depending on sample curvature) and placed 10 cm before the objective. In this configuration, the objective received a diverging beam that is focused beyond its natural working distance. The laser power at the sample in *imaging mode* was set 100 mW after the objective. Laser light reflected by the reference mirror and backscattered from the cornea were combined using a fiber collimator (C1) and directed towards a custom made spectrometer using a polarization maintaining fiber (PMF). The spectrometer simply consisted of a collimator (C2), a transmission diffraction grating (1600 lines/mm), an achromatic lens (AC lens, f=200 mm) and a linear CCD camera (CCD1000, Entwicklungsbuero Stresing, Berlin, Germany) with 2048 pixels and operated at 3 kHz line scan rate. A Fast Fourier transform (FFT) was performed on the spectrometer data to obtain the axial scan of the cornea. The data points in the Fourier transformed data (axial scan) do not have real distance units and thus the system was calibrated in order to obtain the distance per point in the Fourier transform [28]. The calibration of the system was performed by replacing cornea with a mirror and moving it in steps of 100 µm and the corresponding FFT peak position was tracked. Three-dimensional volumetric Fourier-Domain OCT images of the cornea were acquired by raster scanning the cornea using motorized stages. Using these posterior illumination OCT images, the corneal endothelium was segmented by locating the first maxima of the intensity derivative in the axial direction. These values were used to extract the 3D-curvature of the corneal endothelium by fitting a second order polynomial for every two dimensional set of axial scans (B-Scan), and stored for later use during the dissection. 

In *photo-disruption mode*, the lens (RL) was removed from the beam path so that the objective receives a parallel laser beam allowing achieving the minimum possible laser focal volume after the objective. The laser was focused in cornea solely by the objective and the power was increased. The laser power and the pulse duration after the objective were measured to be 400±3 mW and 148±2 fs respectively. The movement of the two-axis motorized stage (Thorlabs, Newton, NJ) holding the cornea and the motorized z-axis holding the objective along the 3D surface determined from the OCT images was controlled using LabView (National Instruments, Austin, TX) so that the focal volume was maintained at a constant user-defined depth from the endothelial surface. The OCT system was calibrated using Optisol.

Corneas unsuitable for transplantation in humans were obtained within 12 hours after death (Quebec Eye Bank, Montreal, QC, Canada), preserved in Optisol-GS at 4°C, and used within two weeks after death [29,30]. The mean ± standard deviation donor age was 71±11 years and the male to female ratio was 1:1.2. The corneas were cut with a 9-mm circular trephine (Weck; Solan Medtronics, Jacksonville, FL) and were placed in petri dishes filled with Optisol, endothelial side down with no applanation. The corneas were trephined so that the height of the central cornea fall within the working distance of the objective. Fixation to the petri dish was not required and laser cuts were performed up to the edge of the corneal buttons. Stromal lamellar dissections were performed with a posterior approach (i.e. illuminating from the endothelial side), focusing the femtosecond laser at different intended depths, either in steps (200 – 150 – 100 - 50 µm) or in a continuous mode, from zero (intraendothelial central photo-disruption) to 200 µm. High-resolution images and thicknesses were obtained later with a spectral radar OCT (Thorlabs). 

Corneal buttons were stained with trypan blue (vital stain) and alizarin red S (Sigma, Oakville, ON, Canada)[31] and photographed (SteREO Discovery V12, Carl Zeiss Canada, Toronto, ON, Canada). Dead endothelial cells appeared in blue and an image analysis algorithm was designed for quantification. The blue component of all pixels of the RGB images was extracted and an automated threshold based on inter-class variance[32] was applied to create binary image where background pixels represented dead cells and foreground pixels, healthy areas. Endothelial integrity was assessed for various dissection depths. Corneal grafts thicker than 50 µm were separated using two fine forceps and the endothelial integrity was assessed after graft separation.

## Results

**Figure 2:**
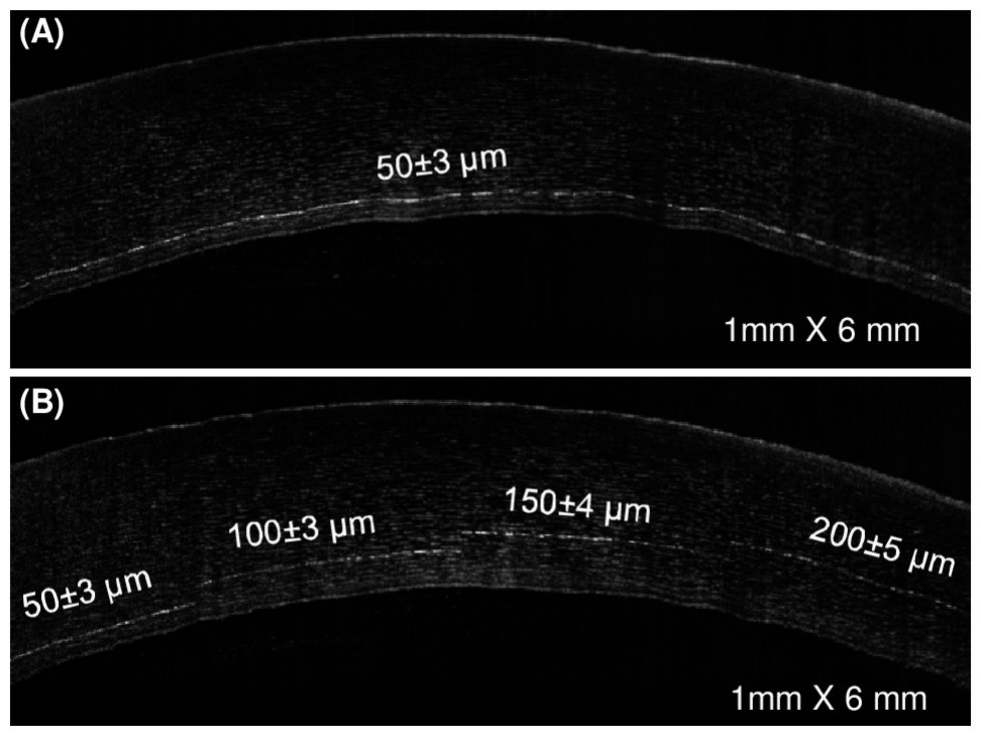
Photo-disruption depth accuracy. (A) The photo-disruption was set at 50 µm from the endothelial surface and the mean (±standard deviation) measured distance was 50±3 µm throughout the 9 mm dissection (B) This micrograph illustrates the ability to vary the photo-disruption depth along the cornea. From left to right, the photo-disruption depth was set at 50 µm, 100 µm, 150 µm, 200 µm from the endothelial surface.

Stromal dissections were performed with this instrument, and their photo-disruption depth accuracy and optimum depth for endothelial cell death was analyzed. Figure 2A shows an OCT image of a 50 μm deep pre-Descemet dissection of a representative corneal sample. 

When 50 μm was set as intended depth, the mean±standard deviation photo-disruption depth obtained was 50±4 microns along the sample (n=9). The sample shown in Figure 2B illustrates our capability to perform dissections at specific arbitrary depths, and even to change it during the procedure. Non-overlapping cuts performed in the same cornea at 50 µm, 100 µm, 150 µm and 200 µm from the endothelial surface are shown. 

**Figure 3:**
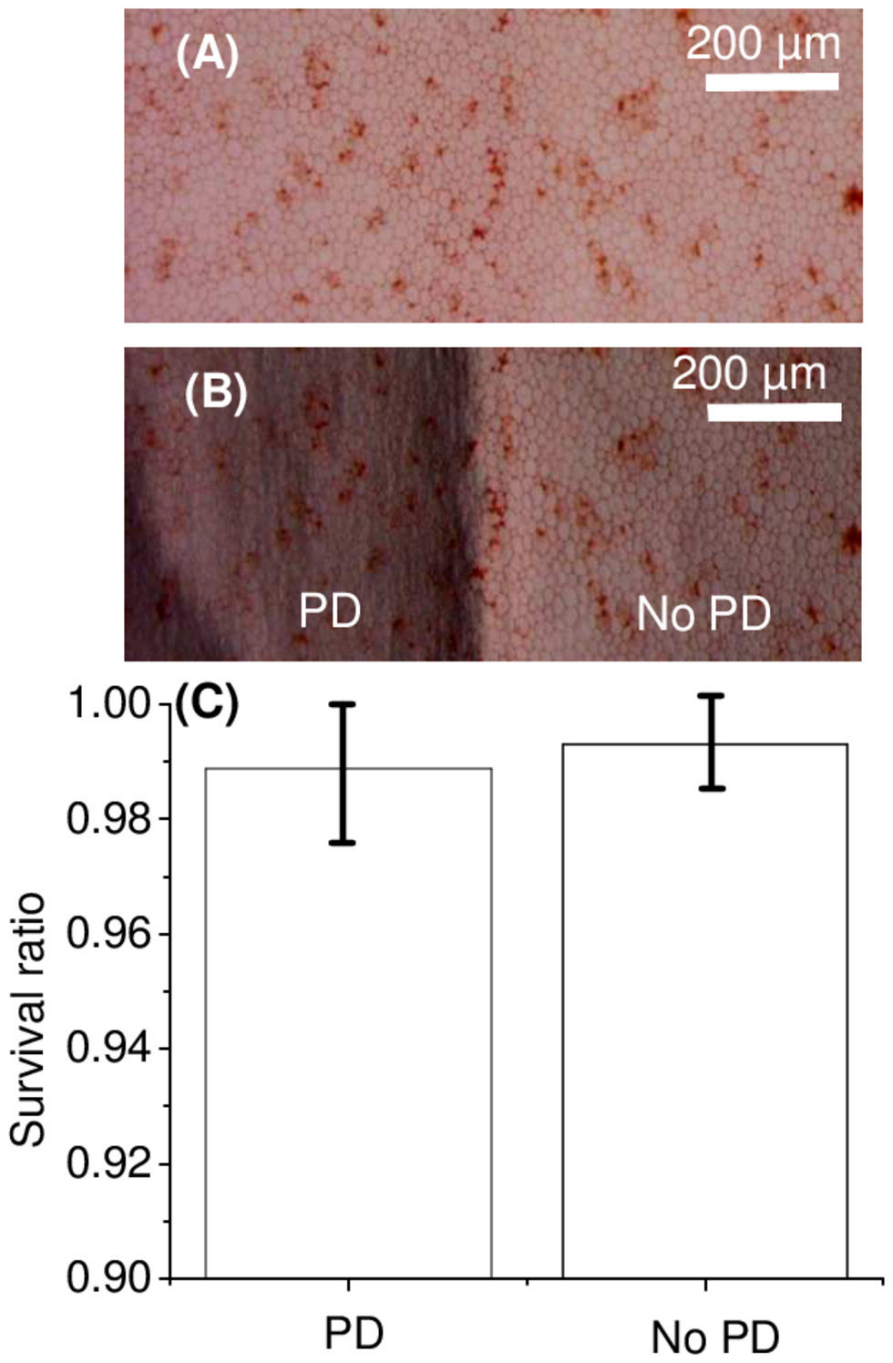
Endothelial cell death above photo-disrupted (PD) and non-photo-disrupted (A) Bright field stereomicroscopy image of the cornea containing both the photo-disrupted and non-photo-disrupted sections. From the image, photo-disrupted and non-photo-disrupted areas can not be identified. (B) Dark field image of the same section of the cornea where focus was adjusted to observe the presence of the photo-disrupted area. (C) The ratio of healthy cell areas to total area analyzed is plotted in the region where we performed the laser treatment at 50 µm depth and an equally big region beside. No significant difference was observed between the two ratios (p=0.34, *t*-test). The data was tested for normality using Shapiro-Wilk test and was found to be with normal distribution.

Endothelial integrity, assessed as described above, revealed no significant differences between laser-treated and untreated regions for a photo-disruption depth of 50 µm. Figure 3 illustrates the comparison between two adjacent regions within each cornea, one treated (1×9 mm strips of photo-disruption at a depth of 50 µm) and the other untreated (n=7 corneas). Similar percentages of healthy endothelial cells were documented in both regions.

Corneal grafts separation after photo-disruption was easy and induced no significant folding or rolling of the grafts after detachment. Measured endothelial cell death after separation was less than 4%. 

**Figure 4:**
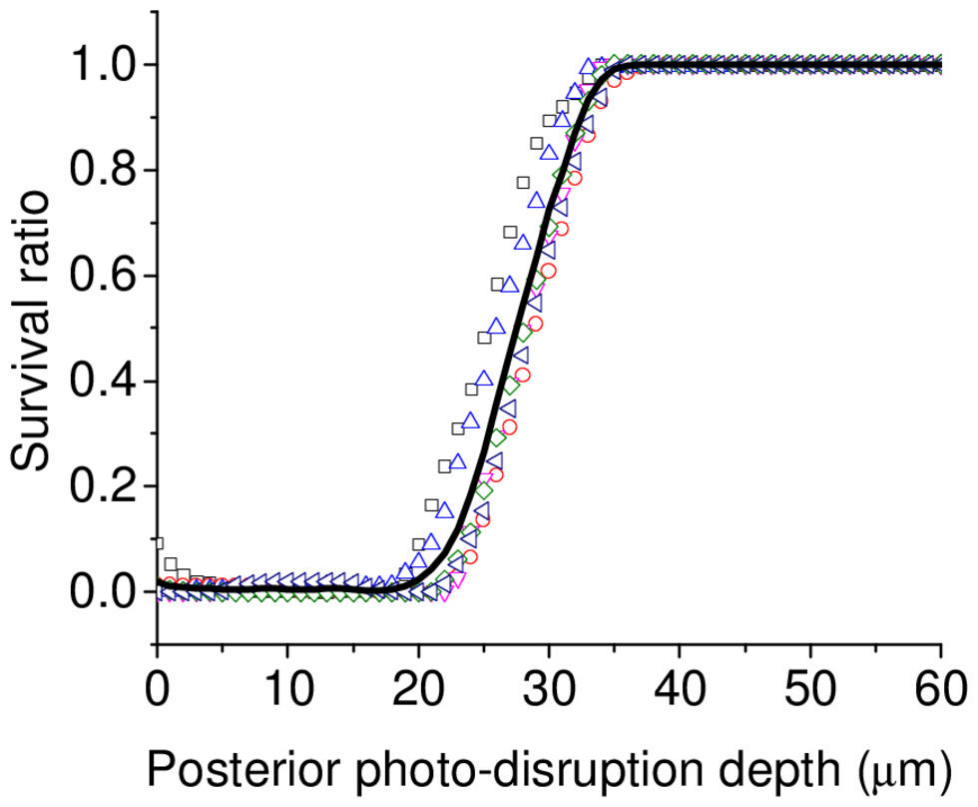
Endothelial cell death as a function of photo-disruption depth. Planar cuts in which we intentionally focused the laser within the endothelial cell layer in the center of cornea were performed. Due to the curvature of the cornea and the planar cut performed, the depth of the photo-disruption was minimum at the center (where cell death is observed) and increased towards the periphery (where no cell death is observed). After dissection, corneas were stained with trypan blue and alizarin red S and photographed. The ratio of the number of pixels in a region of interest that belong to healthy cells to the total number of pixels is plotted. The colored symbols represent the data for six different samples and their average represented by black solid line. The healthy ratio increased to 50% at a photo-disruption depth of 27 µm and to more than 99% at a depth of 34 µm.

One key parameter investigated was the minimum safe photo-disruption depth, i.e. the smallest distance between the endothelial cell layer and the laser focal volume, such that no significant endothelial cell death could be observed. To quantify this, we performed planar (flat) cuts for which we intentionally focused the laser within the endothelial cell layer at the center of cornea. Due to the curvature of the cornea and the planar nature of the cut performed, the depth of the photo-disruption was minimum in the center (laser focussed into the endothelium) and increased towards the periphery. After photo-disruption, corneas were stained and quantified for cell death (n=6) as described above. In this case, a rectangular region of interest (1000×250 µm) was selected (beginning at the center of the cornea where damage to endothelial cells was evident) and displaced in steps of 10 µm towards the periphery, which corresponded to increased dissection depth. The ratio of healthy to total area is shown in Figure 4 for the individual samples and their average. The mean survival ratio was found to increase from 0% for intraendothelial photo-disruption, to 50%, for a photo-disruption depth of 27 µm, and more than 99% at a depth of 34 µm from the posterior surface.

## Discussion and Conclusion

Femtosecond lasers have been widely used in preparation of corneal flaps in LASIK [33] where applanation of the epithelial side of cornea and shallow laser dissections are routinely obtained. Furthermore, they have been widely used for penetrating keratoplasty [34], both for patient and donor tissue [35]. However, this functionality cannot easily be extended to prepare grafts for Endothelial Keratoplasty (EK) [20,36-38]. Firstly, corneal hydration and the subsequent scattering represent a major obstacle for deep penetration of femtosecond pulses, which can hardly be focused as depth increases. This fundamental issue has forced technology developments to switch to more infrared wavelengths for minimizing scattering. This change requires a completely different laser technology, which is not readily available in the market, and despite recent impressive progress in this new field, it is still a laboratory methodology [39]. Secondly, the use of applanation can be problematic. When applied on the epithelium, it creates distortions and ripples in the inner layers of the cornea [40] that would render non-uniform grafts after planar cuts. Furthermore, applanation and contact to the corneal endothelium require greater care, otherwise it can provoke endothelial cell death, which is critical for the post-operative visual outcome and long-term graft survival. 

This new device we propose is based on a non-contact posterior approach; OCT images are utilized to obtain a 3D representation of the sample once placed on the holder. This information allows a uniform dissection at very shallow depths, thus avoiding scattering effects. Furthermore, non-amplified femtosecond lasers focused with high NA optics have proven to yield significantly smaller cavitation bubbles, which is essential to dissect the corneal stroma a few microns away from the fragile endothelial cells. Moreover we showed that the same laser source also provides enough bandwidth for low-coherence interferometry imaging. Using the same laser source for both imaging and dissecting allows perfect matching of focusing, while minimizing calibrations, optical design and cost. 

The thickness of the grafts could be controlled with approximately 5 µm accuracy along 9 mm diameter cuts with our prototype, and no significant damage to the endothelial cells was found for photo-disruption depths beyond 34 µm. Furthermore, we have shown that the dissection depth can be varied within a sample, which allows to tailor the grafts as desired and seek refractive compensation. Previous studies where femtosecond lasers were used for preparing endothelial grafts reported interface haze [21] and a lower visual acuity [20] than that usually seen after penetrating keratoplasty or DSAEK. This can be attributed to the distortion of the inner corneal layers caused by the applanation of the front corneal surface during the cutting procedure [41]. Wound healing of the posterior stromal lamellae after femtosecond laser dissection might also be different from that of the anterior layers. The grafts prepared in our study were not transplanted in patients, precluding our ability to assess postoperative visual acuity.

Overall, our proof-of-concept experiments demonstrate that this instrument could be particularly useful in eye-bank setups where the ability to produce precut tissue of customized shape can render a significant improvement in quality. Hence, grafts of constant thickness within few microns will minimize optical aberrations, thus optimizing visual outcome. 

## References

[1] Price FW Jr., Price MO (2006) Descemet's stripping with endothelial keratoplasty in 200 eyes: Early challenges and techniques to enhance donor adherence. J Cataract Refract Surg 32: 411-418. doi:10.1016/j.jcrs.2005.12.078. PubMed: 16631048. 16631048

[2] Price MO, Price FW Jr. (2006) Descemet's stripping with endothelial keratoplasty: comparative outcomes with microkeratome-dissected and manually dissected donor tissue. Ophthalmology 113: 1936-1942. doi:10.1016/j.ophtha.2006.05.034. PubMed: 16935344. 16935344

[3] Bahar I, Kaiserman I, McAllum P, Slomovic A, Rootman D (2008) Comparison of posterior lamellar keratoplasty techniques to penetrating keratoplasty. Ophthalmology 115: 1525-1533. doi:10.1016/j.ophtha.2008.02.010. PubMed: 18440638. 18440638

[4] Covert DJ, Koenig SB (2007) Descemet stripping and automated endothelial keratoplasty (DSAEK) in eyes with failed penetrating keratoplasty. Cornea 26: 692-696. doi:10.1097/ICO.0b013e31805fc38f. PubMed: 17592318. 17592318

[5] Dupps WJ Jr., Qian Y, Meisler DM (2008) Multivariate model of refractive shift in Descemet-stripping automated endothelial keratoplasty. J Cataract Refract Surg 34: 578-584. doi:10.1016/j.jcrs.2007.11.045. PubMed: 18361978. 18361978 PMC2796246

[6] Holz HA, Meyer JJ, Espandar L, Tabin GC, Mifflin MD et al. (2008) Corneal profile analysis after Descemet stripping endothelial keratoplasty and its relationship to postoperative hyperopic shift. J Cataract Refract Surg 34: 211-214. doi:10.1016/j.jcrs.2007.09.030. PubMed: 18242442. 18242442

[7] Jun B, Kuo AN, Afshari NA, Carlson AN, Kim T (2009) Refractive change after descemet stripping automated endothelial keratoplasty surgery and its correlation with graft thickness and diameter. Cornea 28: 19-23. doi:10.1097/ICO.0b013e318182a4c1. PubMed: 19092399. 19092399

[8] Koenig SB, Covert DJ, Dupps WJ Jr., Meisler DM (2007) Visual acuity, refractive error, and endothelial cell density six months after Descemet stripping and automated endothelial keratoplasty (DSAEK). Cornea 26: 670-674. doi:10.1097/ICO.0b013e3180544902. PubMed: 17592314. 17592314

[9] Price MO, Price FW Jr., Stoeger C, Soper M, Locke GD et al. (2008) Central thickness variation in precut DSAEK donor grafts. J Cataract Refract Surg 34: 1423-1424. doi:10.1016/j.jcrs.2008.05.044. PubMed: 18721690. 18721690

[10] Rao SK, Leung CK, Cheung CY, Li EY, Cheng AC et al. (2008) Descemet stripping endothelial keratoplasty: effect of the surgical procedure on corneal optics. Am J Ophthalmol 145: 991-996. doi:10.1016/j.ajo.2008.01.017. PubMed: 18342831. 18342831

[11] Scorcia V, Matteoni S, Scorcia GB, Scorcia G, Busin M (2009) Pentacam Assessment of Posterior Lamellar Grafts to Explain Hyperopization after Descemet Stripping Automated Endothelial Keratoplasty. Ophthalmology 116: 1651-1655. doi:10.1016/j.ophtha.2009.04.035. PubMed: 19643500. 19643500

[12] Yoo SH, Kymionis GD, Deobhakta AA, Ide T, Manns F et al. (2008) One-year results and anterior segment optical coherence tomography findings of descemet stripping automated endothelial keratoplasty combined with phacoemulsification. Arch Ophthalmol 126: 1052-1055. doi:10.1001/archopht.126.8.1052. PubMed: 18695098. 18695098

[13] Busin M, Patel AK, Scorcia V, Ponzin D (2012) Microkeratome-assisted preparation of ultrathin grafts for descemet stripping automated endothelial keratoplasty. Invest Ophthalmol Vis Sci 53: 521-524. doi:10.1167/iovs.11-7753. PubMed: 22205600. 22205600

[14] Busin M, Madi S, Santorum P, Scorcia V, Beltz J (2013) Ultrathin descemet's stripping automated endothelial keratoplasty with the microkeratome double-pass technique: two-year outcomes. Ophthalmology 120: 1186-1194. doi:10.1016/j.ophtha.2012.11.030. PubMed: 23466268. 23466268

[15] Melles GR, Ong TS, Ververs B, van der Wees J (2006) Descemet membrane endothelial keratoplasty (DMEK). Cornea 25: 987-990. PubMed: 17102683. 17102683 10.1097/01.ico.0000248385.16896.34

[16] Price MO, Giebel AW, Fairchild KM, Price FW Jr. (2009) Descemet's membrane endothelial keratoplasty: prospective multicenter study of visual and refractive outcomes and endothelial survival. Ophthalmology 116: 2361-2368. doi:10.1016/j.ophtha.2009.07.010. PubMed: 19875170. 19875170

[17] Terry MA, Straiko MD, Goshe JM, Li JY, Davis-Boozer D (2012) Descemet's Stripping Automated Endothelial Keratoplasty: The Tenuous Relationship between Donor Thickness and Postoperative Vision. Ophthalmology 119: 1988-1996. doi:10.1016/j.ophtha.2012.05.021. PubMed: 22817831. 22817831

[18] Woodward MA, Raoof-Daneshvar D, Mian S, Shtein RM (2013) Relationship of visual acuity and lamellar thickness in descemet stripping automated endothelial keratoplasty. Cornea 32: e69-e73. doi:10.1097/ICO.0b013e318271fc99. PubMed: 23132458. 23132458

[19] Neff KD, Biber JM, Holland EJ (2011) Comparison of central corneal graft thickness to visual acuity outcomes in endothelial keratoplasty. Cornea 30: 388-391. doi:10.1097/ICO.0b013e3181f236c6. PubMed: 21045647. 21045647

[20] Cheng YYY, Pels E, Nuijts RMMA (2007) Femtosecond-laser–assisted Descemet's stripping endothelial keratoplasty. Journal of Cataract and Refractive Surgery 33: 152-155. doi:10.1016/j.jcrs.2006.07.044. 17189814

[21] Hjortdal J, Nielsen E, Vestergaard A, Søndergaard A (2012) Inverse cutting of posterior lamellar corneal grafts by a femtosecond laser. Open Ophthalmol J 6: 19-22 10.2174/1874364101206010019 PMC3349952 22582107

[22] Di Pascuale MA, Prasher P, Schlecte C, Arey M, Bowman RW, et al. (2009) Corneal deturgescence after Descemet stripping automated endothelial keratoplasty evaluated by Visante anterior segment optical coherence tomography. Am J Ophthalmol 148: 32-37 e31 19327750 10.1016/j.ajo.2009.01.016

[23] Radhakrishnan S, Rollins AM, Roth JE, Yazdanfar S, Westphal V et al. (2001) Real-time optical coherence tomography of the anterior segment at 1310 nm. Arch Ophthalmol 119: 1179-1185. doi:10.1001/archopht.119.8.1179. PubMed: 11483086. 11483086

[24] Singh K, Dion C, Wajszilber M, Ozaki T, Lesk MR et al. (2011) Measurement of Ocular Fundus Pulsation in Healthy Subjects Using a Novel Fourier-Domain Optical Coherence Tomography. Invest Ophthalmol Vis Sci 52: 8927-8932. doi:10.1167/iovs.11-7854. PubMed: 21969303. 21969303

[25] Huang D, Swanson EA, Lin CP, Schuman JS, Stinson WG et al. (1991) Optical Coherence Tomography. Science 254: 1178-1181. doi:10.1126/science.1957169. PubMed: 1957169. 1957169 PMC4638169

[26] Fercher AF, Hitzenberger CK, Kamp G, El-Zaiat SY (1995) Measurement of intraocular distances by backscattering spectral interferometry. Optics Communications 117: 43-48. doi:10.1016/0030-4018(95)00119-S.

[27] Wojtkowski M, Srinivasan VJ, Ko TH, Fujimoto JG, Kowalczyk A et al. (2004) Ultrahigh-resolution, high-speed, Fourier domain optical coherence tomography and methods for dispersion compensation. Opt Express 12: 2404-2422. doi:10.1364/OPEX.12.002404. PubMed: 19475077. 19475077

[28] Singh K, Dion C, Godin AG, Lorghaba F, Descovich D et al. (2012) Pulsatile Movement of the Optic Nerve Head and the Peripapillary Retina in Normal Subjects and in. Journal of Glaucoma - Investigative Ophthalmology and Visual Science 53: 7819-7824. doi:10.1167/iovs.12-9834. 23099495

[29] Jeng BH (2006) Preserving the cornea: corneal storage media. Curr Opin Ophthalmol 17: 332-337. doi:10.1097/01.icu.0000233950.63853.88. PubMed: 16900023. 16900023

[30] Wagoner MD, Gonnah el S (2005) Corneal graft survival after prolonged storage in Optisol-GS. Cornea 24: 976-979. doi:10.1097/01.ico.0000159731.52801.b6. PubMed: 16227845. 16227845

[31] Taylor MJ, Hunt CJ (1981) Dual staining of corneal endothelium with trypan blue and alizarin red S: importance of pH for the dye-lake reaction. Br J Ophthalmol 65: 815-819. doi:10.1136/bjo.65.12.815. PubMed: 6172144. 6172144 PMC1039687

[32] Otsu N (1979) A threshold selection method from gray-level histograms. IEEE Transactions on Systems, Man, and Cybernetics 9: 62-66. doi:10.1109/TSMC.1979.4310076.

[33] Güell JL, Muller A (1996) Laser in situ keratomileusis (LASIK) for myopia from -7 to -18 diopters. Journal of Refractive Surgery (Thorofare, NJ : 1995) 12: 222-228. PubMed: 8653524. 10.3928/1081-597X-19960201-03 8653524

[34] Buratto L, Böhm E (2007) The Use of the Femtosecond Laser in Penetrating Keratoplasty. American Journal of Ophthalmology 143: 737-742. 17368554 10.1016/j.ajo.2007.01.056

[35] Meltendorf C, Schroeter J, Bug R, Kohnen T, Deller T (2006) Corneal Trephination With the Femtosecond Laser. Cornea 25: 1090-1092. 17133060 10.1097/01.ico.0000228784.46463.e9

[36] Monterosso C, Fasolo A, Caretti L, Monterosso G, Buratto L, et al. (2011) Sixty-Kilohertz Femtosecond Laser–Assisted Endothelial Keratoplasty: Clinical Results and Stromal Bed Quality Evaluation. Cornea 30: 189-193. 20885308 10.1097/ICO.0b013e3181ead924

[37] Mehta JS, Shilbayeh R, Por Y-M, Cajucom-Uy H, Beuerman RW et al. (2008) Femtosecond laser creation of donor cornea buttons for Descemet-stripping endothelial keratoplasty. J Cataract Refract Surg 34: 1970-1975. doi:10.1016/j.jcrs.2008.07.028. PubMed: 19006747. 19006747

[38] Ide T, Yoo SH, Kymionis GD, Leng T, Marini C, et al. (2010) Descemet Stripping Automated Endothelial Keratoplasty Tissue Preparation With Femtosecond Laser and Contact Lens. Cornea 29: 93-98. 19907309 10.1097/ICO.0b013e3181967052

[39] Peyrot DA, Aptel F, Crotti C, Deloison F, Lemaire S et al. (2010) Effect of incident light wavelength and corneal edema on light scattering and penetration: laboratory study of human corneas. J Refract Surg 26: 786-795. doi:10.3928/1081597X-20100921-04. PubMed: 20954687. 20954687

[40] Mootha VV, Heck E, Verity SM, Petroll WM, Lakshman N et al. (2011) Comparative study of descemet stripping automated endothelial keratoplasty donor preparation by Moria CBm microkeratome, horizon microkeratome, and Intralase FS60. Cornea 30: 320-324. PubMed: 21304290. 21304290 10.1097/ICO.0b013e3181f22cc3 PMC3569007

[41] Soong HK, Mian S, Abbasi O, Juhasz T (2005) Femtosecond laser-assisted posterior lamellar keratoplasty: initial studies of surgical technique in eye bank eyes. Ophthalmology 112: 44-49. doi:10.1016/j.ophtha.2004.06.037. PubMed: 15629819. 15629819

